# The Protective Effect of α-Lipoic Acid against Gold Nanoparticles (AuNPs)-Mediated Liver Damage Is Associated with Upregulating Nrf2 and Suppressing NF-κB

**DOI:** 10.3390/nu14163327

**Published:** 2022-08-14

**Authors:** Ghedeir M. Alshammari, Mohamed Anwar Abdelhalim, Mohammed S. Al-Ayed, Laila Naif Al-Harbi, Mohammed Abdo Yahya

**Affiliations:** 1 Department of Food Science & Nutrition, College of Food and Agricultural Sciences, King Saud University, Riyadh 11451, Saudi Arabia; 2 Department of Physics and Astronomy, College of Science, King Saud University, Riyadh 11451, Saudi Arabia

**Keywords:** gold nanoparticles, Vitamin E, α-lipoic acid, hepatotoxicity, oxidative stress

## Abstract

This study examined if regulating the keap-1? Nrf2 antioxidant pathway mediated gold nanoparticles (AuNPs) induced liver damage, and examined the protective effect of co-supplement of α-lipoic acid (α-LA). Rats were separated into 4 groups (n = 8/each) as control, α-LA (200 mg/kg), AuNPs (5 µg/2.85 × 10^11^), and AuNPs (5 µg/2.85 × 10^11^) + α-LA (200 mg/kg). After 7 days, AuNPs induced severe degeneration in the livers of rats with the appearance of some fatty changes. In addition, it increased serum levels of alanine aminotransferase (ALT) and gamma-glutamyl transferase (ɣ-GTT), and aspartate aminotransferase (AST), as well as liver levels of malondialdehyde (MDA). Concomitantly, AuNPs significantly depleted hepatic levels of total glutathione (GSH), superoxide dismutase (SOD), and catalase (CAT) but increased hepatic levels of tumor necrosis factor-α (TNF-α) and interleukin-6 (IL-6). It also reduced mRNA levels of B-cell lymphoma 2 (Bcl2) and heme oxygenase-1 (HO-1) but significantly increased those of Bax and cleaved caspase-3, as well as the ratio of Bax/Bcl2. In addition, AuNPs enhanced the total and nuclear levels of NF-κB p65 but reduced the mRNA and total and nuclear protein levels of Nrf2. Of note, AuNPs did not affect the mRNA levels of keap-1. All these events were reversed by α-LA in the AuNPs-treated rats. In conclusion, α-LA attenuated AuNPs-mediated liver damage in rats by suppressing oxidative stress and inflammation, effects that are associated with upregulation/activation of Nrf2.

## 1. Introduction

In recent years, the use of nanomaterials (NM) has dramatically increased in industry, agriculture, and medicine due to their relatively small size (1–100) nm, volume surface area (>60 m^2^/cm), and chemical properties [1]. NMs include nanoplates, nanofibers, and metal nanoparticles (NPs) [1]. Common NPs include gold (AuNPs), titanium dioxide (TiO_2_NPs), cadmium sulphide (CdSNPs), silver (AgNPs), silicon (SiO_2_NPs), cobalt NPs (CoNPs), cerium oxide (CeO_2_NPs), and zinc oxide (ZnONPs) [2]. However, toxicological studies have reported that repetitive exposure to these NPs is associated with multisystem toxicities due to their role in generating high levels of active, reactive oxygen species (ROS), and subsequently promoting oxidative stress and inflammation [1,2,3,4,5].

AuNPs are unique for their shape, size, chemical stability, easy functionalization, and optoelectronic properties [6]. For this reason, AuNPs are largely used in imaging, biosensor manufacturing, gene delivery, gene therapy, cancer treatment, and water purification, which results in a high exposure rate [7]. In experimental animals, exposure to small-size AuNPs (<50 nm) resulted in an accumulation in several organs, whereas >50 nm was mainly detected in the liver and kupffer cells [7,8]. In addition, treatment with small AuNPs is associated with renal and hepatic oxidative damage and toxicities [7,9]. Besides, AuNPs exaggerated hepatic damage after exposure to lipopolysaccharides (LPS) and choline-deficient diet-induced hepatic steatosis [10,11]. At the biochemical levels, AuNPs-mediated hepatotoxicity was associated with high ROS and inflammatory cytokines, low antioxidant expression, and increased activation of the nuclear factor kappa-beta (NF-κB p65), a master inflammatory transcription factor [8,11,12,13,14]. Despite this, the precise mechanisms underlying the toxicological profile of AuNPs are still unknown.

The nuclear factor erythroid-derived 2-like 2 (Nrf2) is the major antioxidant transcription factor in the cell that stimulates the transcription of antioxidant genes via the binding to the antioxidant response element (ARE) [15,16]. Additionally, Nrf2 is a potent anti-inflammatory and anti-apoptotic factor due to its ability to downregulate the NACHT, LRR, and PYD domains-containing protein 3 (NRLP3) inflammasome, inhibit the activation of NF-κB and upregulate Bcl2 [17,18,19,20]. The Kelch-like ECH-associated protein 1 (keap-1) is a cysteine-rich protein that normally binds and degrades Nrf2 in the cytoplasm [17]. Under stress, ROS oxidizes keap-1, thus allowing Nrf2 to translocate to the nucleus [20,21]. The pharmacological activator of Nrf2 can also react directly with the thiol group of the cysteine residues of keap-1 and inhibit it [21]. Of note, the activation of Nrf2 is an effective therapy to alleviate hepatic damage, inflammation, and fibrosis in various disorders [22]. In addition, mesoporous silica NPs (MSiNPs), AgNPs, and TiO2NPS promoted liver and kidney oxidative damage and inflammation by suppressing keap-1/Nrf2 signaling [23,24,25]. On the contrary, AuNPs stimulated Nrf2 in cultured endothelial cells, keratinocytes, and caco cells [26,27,28,29]. However, the effect of AuNPs on the hepatic expression/activities of the keap-1/Nrf2 axis in vivo was never shown before, representing a novel target.

Natural antioxidants in edible or medicinal plants are an excellent strategy for treating hepatotoxicity and liver disorders [30]. Additionally, some of these known antioxidants alleviated hepatic damage after intoxication with NPs [31]. Alpha-lipoic acid (α-LA) is one of the most known antioxidants dithiols that can be synthesized in the liver mitochondria or received from meat and vegetables (i.e., tomato, broccoli, and spinach) [31,32]. The antioxidant effect of α-LA is well documented in the liver and other tissue of numerous animal models [31,33,34,35]. In this view, the antioxidant potential of α-LA was attributed to its ability to act via several pathways, including ROS scavenging, chelating metals, upregulation of antioxidants, suppression of NF-κB/inflammatory cytokine axis, and recycling of vitamin E and C. In addition, α-LA is a potent inducer of Nrf2, a major mechanism that underlies its reno, hepatic, cardiac, pulmonary, and neural antioxidant protective effects [32,36,37,38,39].

Interestingly, treatment with α-LA also attenuated MSiNPs-induced neurotoxicity and AgNPs-mediated hepatotoxicity by scavenging ROS [40,41]. Additionally, it alleviated ferroptosis-like cell death in CoNPs-treated Balb/3T3 cells, as well as CoNPs-induced hepatic apoptosis in rats by reducing ROS and lipid peroxides levels and stimulating the total levels of reduced glutathione (GSH) [42,43]. In the same line, α-LA prevented lead (Pb) NPs and ZnNPs-induced neural and reproductive toxicity by chelating potential and suppressing ROS and inflammatory cytokines production [42,44,45,46]. Concomitant administration of vitamin E and α-LA prevented AuNPs-induced renal damage by suppressing ROS and MDA and concomitant upregulation of GSH [9].

However, as discussed above, the wide use of AuNPs in drug delivery and cancer therapy has increased the risk of developing hepatic toxicity. The precise mechanism by which AuNPs promote liver damage is not entirely identified, which we propose to be mediated through modulating Nrf2/antioxidant axis. If proven to be correct, Nrf2 activators may present novel protective agents. Hence, this study was conducted with two major objectives. First, to examine the effect of AuNPs on the expression/activity of keap-1/Nrf2 components in the livers of intoxicated rats, and second, to examine whether co-administration α-LA could alleviate AuNPs-associated hepatic oxidative stress, inflammation, and apoptosis by targeting this axis.

## 2. Materials and Methods

### 2.1. Drugs and Doses

In this study, we used 10 nm GNPs (product No. MKN-Au-010; MK IPEX Corp) to induce oxidative hepatotoxicity, as confirmed in our previous studies after an intraperitoneal (i.p.) daily administration at a concentration of 50 μL for 7 days [7,8,13]. The 50 µL of the 10 nm AuNPs contain an effective concentration of Au of 5 µg and contain 2.85 × 10^11^ of AuNPs. The shape (spherical) and mean size of GNPs were identified using transmission electron microscopy (TEM). α-LA (product No. 62320) was purchased from Sigma Aldrich, Cambridge, UK). Soyabean oil was always dissolved freshly in 0.9% normal saline as a vehicle [46]. The dose of α-LA was given i.p. final doses of 200 mg/kg, a dose that prevented AuPNs-induced kidney damage, Zn-NPs-induced liver damage, and non-alcoholic fatty liver disease in rats if given orally or i.p. [9,44,47]. This also prevented high-fat diet (HFD)-induced hepatic steatosis in mice by upregulation of Nrf2 [48].

### 2.2. Animals

Twenty-four healthy Wistar male rats 12 weeks old, weighing between 220–240 gm, were obtained from the Experimental Animal Care Center at King Saud University, Riyadh, K.S.A. Animals were contained under environmentally controlled conditions (22 ± 5 °C, 55 ± 5% humidity) and a 12 h light/dark cycle. The rats had free access to water and were maintained on a standard rat diet [1]. All experimental protocols included in this study were approved by Research Ethics Committee at King Saud University (Ethics Reference No: KSU-SE-21-11), Riyadh, Saudi Arabia, and all protocols were conducted according to the Animal Research Reporting of in vivo Experiments (ARRIVE) guidelines.

### 2.3. Experimental Design

**Figure 1:**
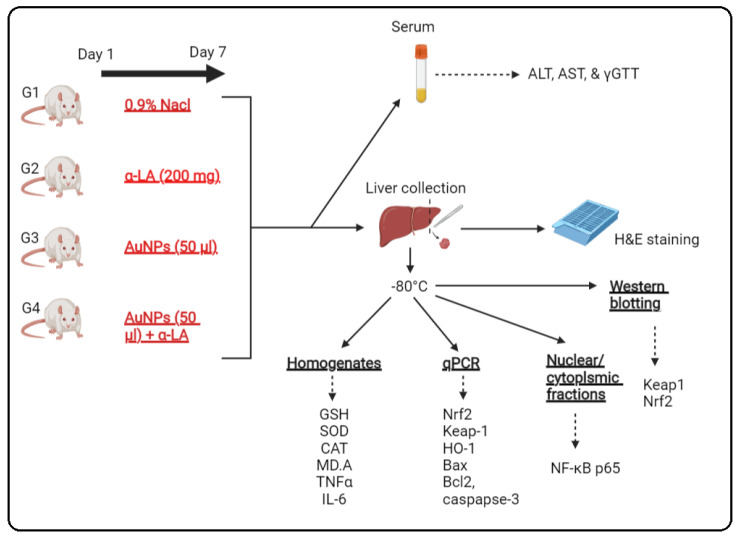
A schematic diagram for the experimental setting used in the current study.

Thirty-two rats were randomly segregated into four treatment groups (n = 8/each) as the following: (1) control rats: i.p., administered 250 µL of 0.9% normal saline as a vehicle; (2) α-LA-treated rats: administered α-LA solution (200 mg/kg) in a final volume of 250 µL; (3) AuNPs-treated rats: were i.p. administered with 50 μL of 10 nm AuNPs (containing 5 µg Au and 2.85 × 10^11^ AuNPs) and concomitantly received 250 µL of 0.9% normal saline (i.p); (4) AuNPs + α LA-treated rats: administered 50 μL of 10 nm AuNPs and concomitantly received 250 µL of α-LA (200 mg/kg). All treatments were administered i.p. for consecutive 7 days daily. Then, the below procedures were conducted and summarized in schematic Figure 1.

### 2.4. Serum and Liver Collection

Twelve hours after the last treatment on day 7, all rats were anesthetized by ketamine/Xylazine hydrochloride mixture (80/10 mg/kg, *v*:*v*). Blood samples (2 mL) were directly collected from the heart of each rat and placed in serum collection plain tubes. All tubes were centrifuged at 1100× *g* for 10 min to collect serum. All serum samples were aliquoted and stored at −20 °C. The serum levels of alanine aminotransferase (ALT) and gamma-glutamyl transferase (ɣ-GTT) were measured by rat’s special ELISA kits (Product No. MBS269614 and Product No. MBS9343646, MyBioSource, San Diego, CA, USA). Serum levels of aspartate aminotransferase (AST) were also measured by an ELISA rats’ specialized kit (Product No. CSB-E13023r-1, Cosmo Bio, Carlsbad, CA, USA). All analyses were conducted for n = 10 samples/group as per each kit’s instructions. The absorbance of each plate was read using SpectraMax Plus Microplate Reader (S/N: 02,927 SoftMax Pro 5; Molecular Devices, San Jose, CA, USA) at 450 mm.

### 2.5. Liver Collection and Processing

After blood collection, all rats were ethically euthanized by the neck dislocation, and their abdomens were opened. The livers were rapidly removed on ice and washed with ice-cold phosphate-buffered saline (PBS) (pH = 7.4) to remove any blood. Livers were cut into smaller cuboidal sections. Parts of these sections were directly fixed in 10% buffered formalin and used within 20 h for histological studies. All other parts were snap-frozen in liquid nitrogen and preserved at −80 °C for biochemical analysis. Later, parts of the frozen livers were homogenized in ice-cold PBS or radioimmunoassay precipitation buffer (RIPA) (Product No. MBS842826, MyBioSource, CA, USA) (plus protease inhibitors (Product No. A32963, ThermoFisher, Waltham, MA, USA) and centrifuged at 12,000× *g* to isolate supernatants which represent total cell homogenates or total protein extracts, respectively. In addition, the cytoplasmic and nuclear fractions of the livers of all groups of rats were prepared using the NE-PER commercial kit (Product No. 78833, ThermoFisher, Waltham, MA, USA). Supernatants and all isolated fractions were maintained at −80 °C until analysis.

### 2.6. Biochemical Analysis in the Liver Homogenates and Isolated Fractions

A colorimetric-based kit was used to assess the homogenate levels of malondialdehyde (MDA) (a lipid peroxidation marker) (Product No. 10009055, Cayman, Ann Arbor, MI, USA). The levels of interleukin-6 (IL-6) and total glutathione (GSH) were measured by enzyme-linked immunosorbent assay (ELISA) kits (Product No. R6000B, R&D System, Minneapolis, MN, USA), Product No. orb782371, Biorbyt, St Louis, MO, USA). The homogenate levels of superoxide dismutase (SOD), catalase (CAT), tumor necrosis factor-alpha (TNF-α), keap-1, and total/nuclear NF-κB p65 were assayed also by ELISA (Product No. MBS036924, Product No. MBS006963, Product No. BMS622, Product. No. MBS7218529; Product No. MBS2505513, MyBioSource, CA, USA, respectively). All protocols were conducted per each kit’s instructions for n = 8 samples/group and following the manufacturer’s recommendations and instructions. Absorbance was read using SpectraMax Plus Microplate Reader S/N: 02927 SoftMax Pro 5; Molecular Devices, CA, USA.

### 2.7. Real-Time Polymerase Chain Reaction (qPCR)

Real-time PCR was conducted to measure the mRNA transcript levels of Keap-1, Nrf2, B-cell lymphoma 2 (Bcl2), Bax, caspase-3, and β-actin (a reference gene). All primers were provided by ThermoFisher and were previously used by us and others [49,50]. The total RNA was isolated using the TRIZOl reagent, where the first-strand cDNA was synthesized using a commercial kit (Product No. GE27-9261-01, Roche Diagnostic company, Indianapolis, IN, USA). All amplifications were carried out using the CFX69 real-time PCR machine (Biorad) and as per the amplification steps provided by the Sofas Evergreen master mix kit (# 172-5200, Biorad, Hercules, CA, USA). In brief, the total amplification volume was 20µL/well, containing the following ingredients: 2 μL cDNA (50 ng/well); 10 µL of the master mix reagent; 0.2 µL of the forward primer (500 nM/each), 0.2 µL of reverse primer (500 nM/each), and 7.6 µL nuclease-free water. Steps of the amplifications are heating (1 cycle/98 °C/30 s; denaturation (40 cycles/98 °C/5 s), annealing (40 cycles/60 °C/5 s), and melting (1 cycle/5 s/60–95 °C). The relative expression of all targets was normalized to the expression of the reference gene, β-actin.

### 2.8. Immunoblotting

The total cytoplasmic and nuclear proteins were prepared in the loading dye buffer to a final concentration of 2 µg/µL. All tubes were then boiled at 100 °C for 5 min. Equal protein concentrations (60 µg/well) were separated by SDS-PAGE, transferred to a nitrocellulose membrane, and then incubated with the primary antioxidants against keap-1 (Product No. 4678, 60 kDa), Nrf2 (Product No. 12721, 100 kDa), β-actin (Product No. 4970, 45 kDa), and lamine A (nuclear loading control) (Product No. 86846, 74 kDa) (all from Cell Signaling Technology, Danvers, MA, USA). The membranes were then washed with the washing buffer and incubated with the HRP peroxidase-conjugated 2nd antibody. The developed bands were scanned, visualized, and photographed using the C-Di Git blot scanner (LI-COR, Lincoln, NE, USA) and its provided software after incubating each membrane with the chemiluminescence west-pico reagent (Cat. No. 34580, Thermo Fisher, U.S.A., Piscataway, NJ, USA).

### 2.9. Hematoxylin and Eosin (H & E) Staining

Formalin preserved livers were deparaffinized in xylene and then with reduced levels of 100%, 90%, and 70%. After this, all samples were embedded in wax, cut in a rotatory microtome (3–5 µm), and stained with Harris hematoxylin/glacial acetic acid solution. Next, the samples were de-stained with 1:400 *v*/*v* HCL/ethanol (70%) solution and stained with one drop of eosin. All samples were then dehydrated with ethanol and xylene and covered with a mounting media and a coverslip. The next day, all tissue was examined under a light microscope and photographed at 200×.

### 2.10. Statistical Analysis

GraphPad Prism analysis software (Version 8) was used for the statistical analysis of all data. Kolmogorov-Smirnov test was utilized to test the normality. Analysis was performed using the 1-way ANOVA test. The levels of significance were determined using Tukey’s test as post hoc (*p* < 0.05). All data were expressed in the results as means ± standard deviation (SD).

## 3. Results

### 3.1. α-LA Prevents the Increase in Liver Function Enzymes in the Serum of AuNPs-Treated Rats

**Figure 2:**
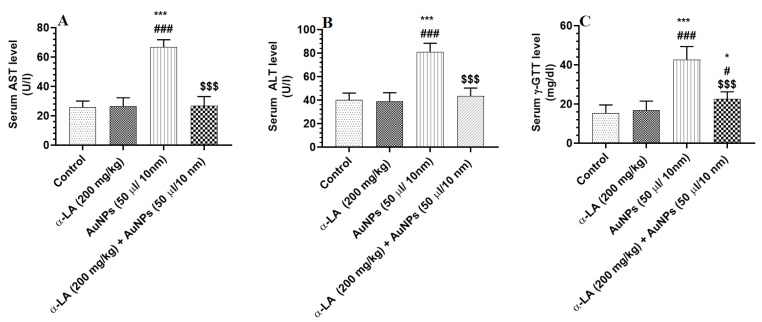
α-lipoic acid (LA) reduces serum levels of liver enzymes in gold nanoparticles (AUNPs)-treated rats. (**A**–**C**): serum levels of aspartate aminotransferase (AST), alanine aminotransferase (ALT), and gamma-glutamyl transferase (ɣ-GTT) (**C**) in treated groups. All measurements were performed by ELISA. Data were given as means ± SD (n = 8/group). *p* < 0.05. *, ***: vs. control at *p* < 0.01 and 0.0001, respectively; #, ###: vs. α-LA at *p* < 0.1 and 0.0001, respectively; and $$$: vs. AuNPs at *p* < 0.0001.

Serum levels of ALT, AST, and γ-GTT were not significantly different in α-LA-treated rats but were significantly increased in the serum of AuNPs-treated rats as compared to control rats (Figure 2A–C). The levels of all these markers were significantly reduced in the serum of AuNPs + α-LA, as compared to AuNPs-treated rats (Figure 2A–C). While serum levels of ALT and AST were not statistically varied, the levels of γ-GTT remained significantly higher when AuNPs + α-LA-treated rats were compared with control rats (Figure 2A–C).

### 3.2. α-LA Stimulates Endogenous Antioxidants in the Livers of Control and AuNPs-Treated Rats

**Figure 3:**
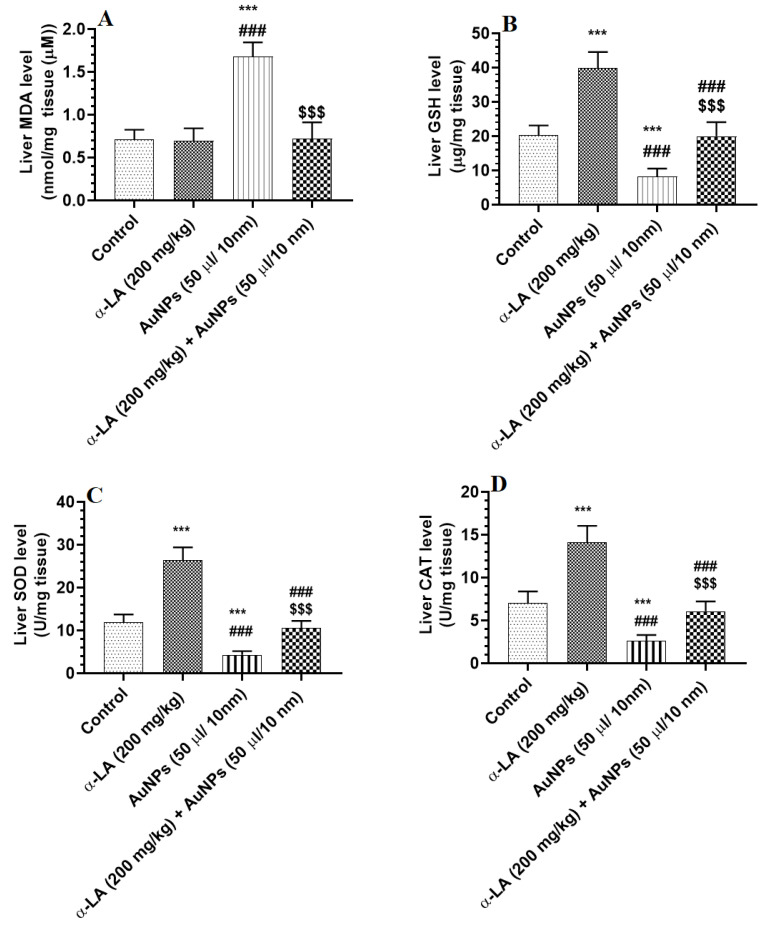
α-lipoic acid (LA) suppresses lipid peroxidation in the livers of gold nanoparticles (AUNPs)-treated rats but stimulates endogenous antioxidants in the livers of the control and AUNPs-treated rats. (**A**): malondialdehyde (MDA) (lipid peroxides), (**B**): total glutathione reduced (GSH), (**C**): superoxide dismutase (SOD), and (**D**): catalase (CAT). Measurements of MDA were performed by an assay kit, and the measurements of all other antioxidants were performed by ELISA. Data were given as means ± SD (n = 8/group). *p* < 0.05. ***: vs. control at *p* < 0.0001; ###: vs. α-LA at *p* < 0.0001; and $$$: vs. AuNPs at *p* < 0.0001.

The hepatic levels of MDA were not significantly different between the control and α-LA-treated rats (Figure 3A). Hepatic levels of GSH, SOD, and CAT were significantly increased in α-LA-treated rats as compared to control rats (Figure 3A–D). Hepatic levels of MDA were significantly increased, but hepatic levels of GSH, SOD, and CAT were significantly decreased in AuNPs-treated rats as compared to control rats and were reversed in the livers of AuNPs + α-LA-treated rats (Figure 3A–D). No significant variations in the levels of all these parameters were seen between the control and AuNPs + α-LA-treated rats (Figure 3A–D).

### 3.3. α-LA Suppresses Hepatic Inflammation in AuNPs-Treated Rats

**Figure 4:**
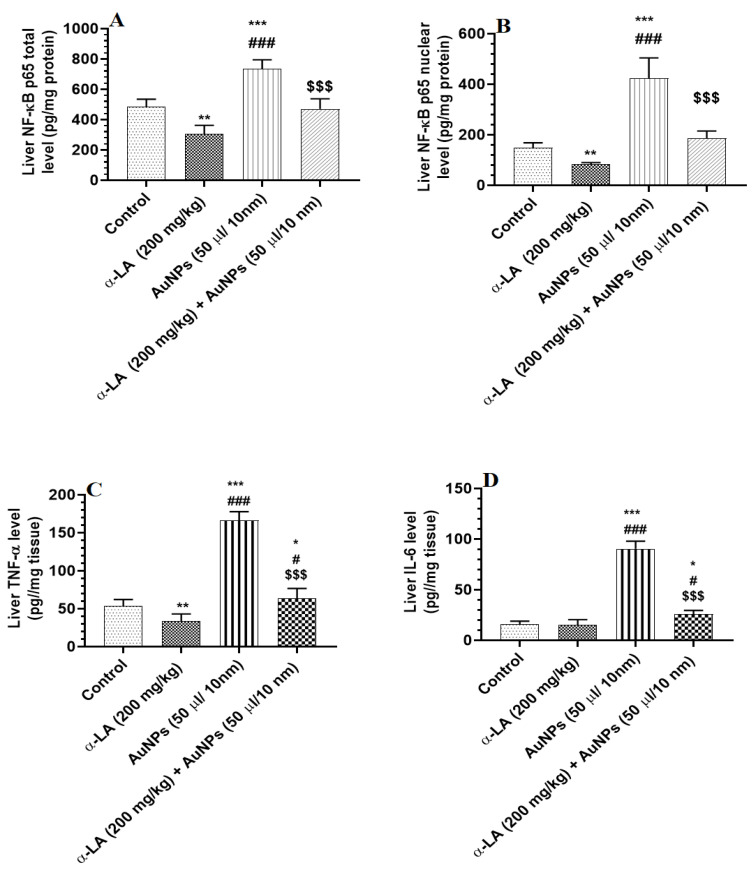
α-lipoic acid (LA) inhibits markers of cellular inflammation in the liver of the control and gold nanoparticles (AUNPs)-treated rats. (**A**,**B**) total and nuclear levels of nuclear factor kappa-beta p65 (NF-κB p65), respectively), (**C**): levels of tumor necrosis factor-alpha (TNF-α), and (**D**): levels of interleukin-6 (IL-6) in the livers of rats of all treated groups. All measurements were conducted by ELISA. Data were given as means ± SD (n = 8/group). *p* < 0.05. *, **, ***: vs. control at *p* < 0.01, 0.001, and 0.0001, respectively; #, ###: vs. α-LA at *p* < 0.01 and 0.0001, respectively; and $$$: vs. AuNPs at *p* < 0.0001.

Total and nuclear levels of NF-κB p65, as well as total levels of TNF-α and IL-6, were significantly increased in the livers of AuNPs-treated rats as compared to control rats (Figure 4A–D). Except for IL-6, the levels of all three inflammatory markers were also reduced in the livers of α-LA-treated rats as compared to control rats (Figure 4A–D). On the other, total and nuclear levels of NF-κB p65, as well as total levels of TNF-α and IL-6, have significantly reduced in the livers of AuNPs + α-LA-treated rats as compared to AuNPs-treated rats (Figure 4A–D). Among all measured markers in AuNPs + α-LA-treated rats, only levels of TNF-α and IL-6 remained slightly higher than their basal levels measured in the control rats (Figure 4A–D).

### 3.4. α-LA Increases Total and Nuclear Levels of Nrf2 in the Livers of Both the Control and AuNPs-Treated Rats

**Figure 5:**
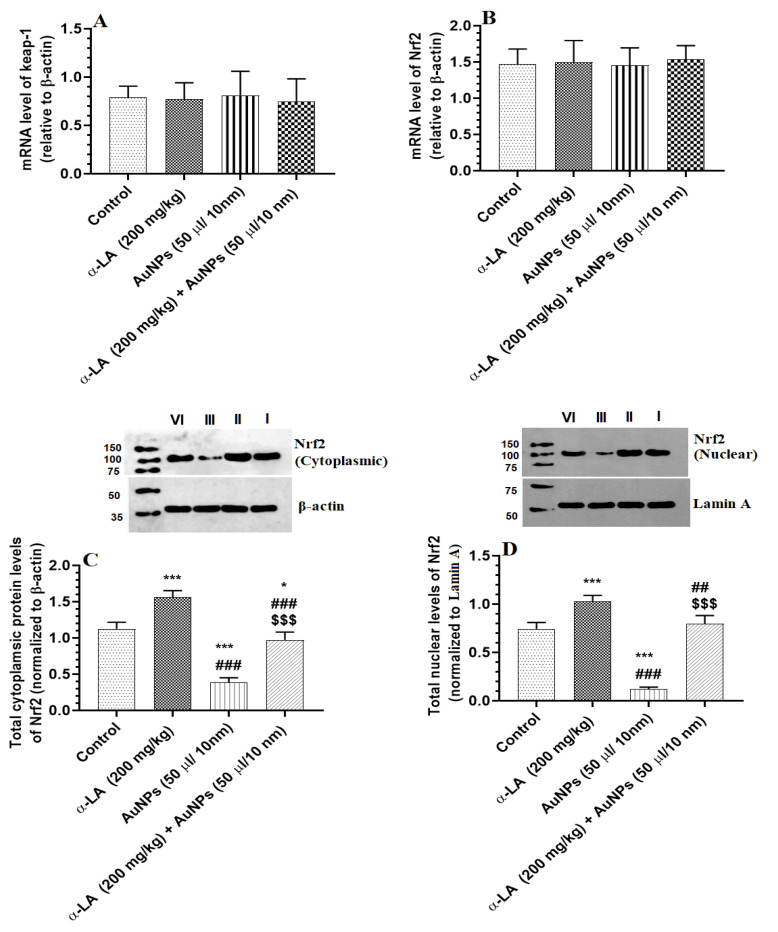
α-lipoic acid (α-LA) increases the total and nuclear protein levels of nuclear factor erythroid-derived 2-like 2 (Nrf2) without altering the mRNA levels of Nrf2 and Kelch-like ECH-associated protein 1 (keap-1) in the livers of the control and gold nanoparticles (AUNPs)-treated rats. (**A**,**B**): mRNA levels of keap-1 and Nrf2, respectively, as detected by real-time PCR. (**C**,**D**): blots and figures representing the total and nuclear levels of Nrf2 as depicted by western blotting. Data were given as means ± SD (n = 8/group) for mRNA levels. *p* < 0.05. *, ***: vs. control (lane I) at *p* < 0.01, and 0.0001, respectively; ##, ###: vs. α-LA (lane II) at *p* < 0.01 and 0.0001, respectively; and $$$: vs. AuNPs (lane III) at *p* < 0.0001. lane IV: AuNPs + α-LA.

Hepatic mRNA levels of keap-1 and Nrf2 were not significantly varied between all groups of rats (Figure 5A,B). The total and nuclear protein levels of Nrf2 were significantly reduced in the livers of AuNPs-treated rats as compared to control rats (Figure 5C,D). A significant increment in the total and nuclear hepatic protein levels of Nrf2 was seen in α-LA-treated and AuNPs + α-LA-treated rats as compared to control or AuNPs-treated rats, respectively (Figure 5C,D). No significant differences in the nuclear protein levels of Nrf2 were seen between the control and AuNPs + α-LA-treated rats (Figure 5C,D).

### 3.5. α-LA Stimulates the Transcription of Bcl2 and HO-1 in the Livers of Control AuNPs-Treated Rats but Downregulates Bax and Caspase-3 in the Livers of AuNPs-Treated Rats

**Figure 6:**
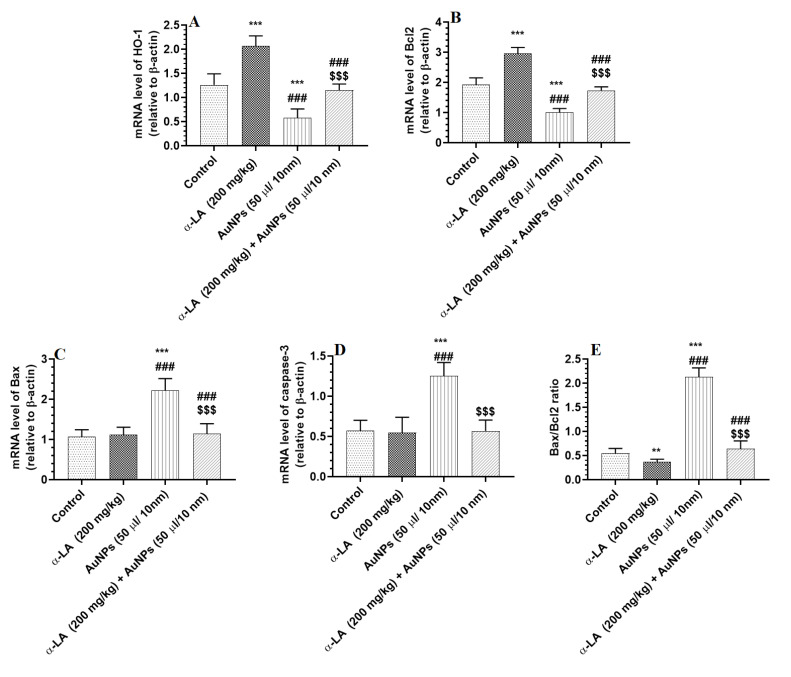
α-lipoic acid (LA) stimulates the transcription of heme-oxygenase-1 (HO-1) (**A**) and Bcl2 (**B**) in the livers of control and gold nanoparticles (AUNPs)-treated rats but suppresses the transcription of Bax (**C**) and caspase-3 (**D**), only, in the livers of AuNPs-treated rats. (**E**): represents the ratio of mRNA levels of Bax to mRNA of Bcl2. Bcl2: an anti-apoptotic protein. Bax and cleaved caspase-3 are Apoptotic proteins. All transcriptions were performed by qPCR. Data were given as means ± SD (n = 8/group). *p* < 0.05. **, ***: vs. control at *p* < 0.001, and 0.0001, respectively; ###: vs. α-LA at *p* < 0.0001, respectively; and $$$: vs. AuNPs at *p* < 0.0001.

mRNA levels of Bax and caspase-3 were significantly increased, but mRNA levels of Bcl2 and HO-1 were significantly reduced in the livers of AuNPs-treated rats as compared to control rats (Figure 6A–D). Higher mRNA levels of HO-1 and Bcl2 parallel with a significant reduction in the Bax/Bcl2 ratio were seen in the livers of α-LA-treated rats as compared to control rats (Figure 6A,D). Additionally, higher mRNA levels of Bcl2 and HO-1 that coincided with lower mRNA levels of Bax and caspase-3, as well as a lower ratio of Bax/Bcl2, have been seen in the livers of AuNPs + α-LA-treated rats as compared to control rats (Figure 6A–D). The mRNA levels of all these targets and the ratio of Bax/Bcl2 were similar between the control and AuNPs + α-LA-treated rats (Figure 6A–D).

### 3.6. α-LA Attenuated Hepatocytes Vacuolization in AuNPs-Treated Rats

**Figure 7:**
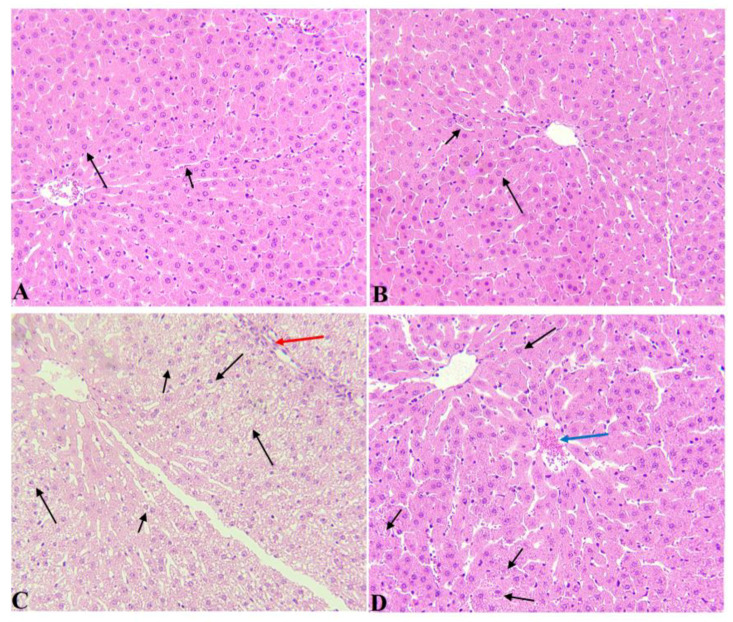
Histological images from all groups of rats. Hematoxyline and eosin (H & E) (200×). (**A**,**B**): were taken from control and α-LA-treated rats and showed normal hepatocytes (long arrow) that radiate from a normally appearing central vein with intact sinusoids (short arrow). (**C**): was taken from AuNPs-treated rats and showed severe hepatocytes degeneration and vacuolization that seemed to be due to cytoplasmic accumulation of lipids (fatty changes). Many nuclei appeared pyknotic (short arrow), and there was obvious immune cell infiltration (red arrow). (**D**): was taken from AuNPs-treated rats and showed much improvement in the structure of the hepatocytes that appeared normal with normal nuclei (Long arrow). Cytoplasmic vacuolization was still seen in a few hepatocytes (short arrow), and there was a loss of hepatocytes (blue arrow).

Livers of the control and α-LA-treated rats showed normal hepatocytes radiating from the central vein (CV) with intact sinusoids (Figure 7A,B). Livers obtained from AuNPs-treated rats showed severe hepatocytes degeneration, increased cytoplasmic vacuolization, immune cell infiltration, and a high number of pyknotic nuclei (Figure 7C). On the other hand, the livers collected from AuNPs-treated rats showed a great deal of improvement in the structure of the hepatocytes that appeared normal with normal nuclei. However, little cytoplasmic vacuolization and hepatocyte loss are still seen (Figure 7D).

## 4. Discussion

The data presented in this study confirm the hepatotoxic effect of repetitive short-term AuNPs in rats by provoking oxidative stress and inflammation. In addition, it demonstrates that the hepatotoxic effect of these particles involves suppression of the keap-1/Nrf2 axis and concomitant increase in the total and nuclear levels of NF-κB p65. On the other hand, this study identifies α-LA as an effective therapeutic option that could alleviate this hepatic toxicity by reversing these events and stimulating the transcriptional activity of Nrf2.

The toxic effects of some NPs, including AuNPs, are mediated by generating high levels of ROS mediated by damaging the mitochondria and promoting endoplasmic stress (ER) stress [1,2,5]. In this study, i.p. treatment of the rats with 50 µL AuNPs (10 nm/2.85 × 10^11^ NPs) induced liver damage and severe hepatocyte vacuolization that was associated with increased serum levels of a major hepatic enzyme (ALT, AST, and GTT), indicating hepatocyte necrosis. In addition, it significantly raised the hepatic levels of ROS and MDA, which coincided with a significant reduction in the levels of GSH, SOD, and CAT. Similar to these data, several previous reports have also shown that treatment with small-sized AuNPs (10 nm and 50 nm) caused severe hepatic damage that was characterized by hepatocyte swelling and necrosis and was associated with increased lipid peroxides levels (i.e., MDA), reduced levels of GSH, and a significant increase in the levels of ALT, GTT, and AST. [7,8,9,13,51,52]. Additionally, these AuNPs exaggerate hepatic damage after administration of LPs or chronic feeding on H.F.D. by stimulating ROS generation [10,11]. On the contrary, these events were prevented by co-treatment with α-LA, thus providing the first evidence for the ability of this molecule to alleviate hepatotoxicity associated with AuNPs. In addition, α-LA reduces the generation of ROS and MDA and stimulates GSH, SOD, and CAT in the livers of control rats, which indicates its ability to upregulate antioxidants and scavenge ROS. Similar antioxidant effects of α-LA were also reported in numerous tissues of several other animal models and were attributed to its ability to scavenge ROS, improve mitochondria function, upregulate antioxidants (e.g., HO-1, CAT, and SOD), reduce the posttranslational O-GlcNAcylation of SOD and CAT, chelate iron, increase cysteine uptake from the plasma (synthesis of GSH), and the recycling GSH and vitamins C and E [53,54,55,56].

Inflammation and mitochondria-mediated (intrinsic) cell apoptosis are hallmarks associated with oxidative stress-induced hepatic damage [57] and were also reported to occur with intoxication with heavy metals [2]. A vicious cycle between ROS and inflammatory cytokines is well-reported [58,59,60]. In addition, a positive association is reported between ROS and NF-κB, which leads to sustained oxidative stress and inflammation [59]. However, ROS and inflammatory cytokines can promote both intrinsic and extrinsic cell death cell apoptosis by several mechanisms which are explained in excellent reviews and studies [61,62,63,64]. Herein, hepatic inflammation and intrinsic apoptosis were also evidenced in the livers of AuNPs-treated rats and were associated with higher levels of total and nuclear levels of Nf-κB p65, levels of TNF-α, and IL-6, Bax, caspase-3, and the ratio of Bax/Bcl2. This effect could be explained by the high ROS generated by AuNPs [61]. These data support those findings reported previously by Kassab et al. [51], who have also shown that naked AuNOs (10–15 nm) induce liver damage by upregulating Bax, downregulating Bcl2, and increasing levels of TNF-α. In addition, AuNPs (10 nm and 50 nm) stimulated hepatic and renal levels of IL-6, TNF-α, and IL-1β in rats [7]. Additionally, naked AuNPs and mainly through a ROS-dependent mechanism, AuNPs stimulated hepatic levels of TNF-α and monocyte chemoattractant protein (MCP-1) in HFD-fed rats as compared to HFD-fed rats alone [10]. In addition, a mixture of Ag and Au NPs significantly increased the activities of NF-κB in HepG2 cells [65]. Additionally, treatment with polyethylene glycol-coated AuNPs (13 nm) resulted in a 7-fold increase in the hepatic activity of NF-κB from 3 h post-administration to day 7 [14]. Furthermore, the apoptotic effect of AuNPs was also demonstrated in the livers of LPS-treated animals or cultured hepatocytes [66].

On the other hand, treatment with α-LA significantly attenuated AuNPs induced hepatic inflammation and apoptosis by reducing levels of TNF-α, downregulating NF-κB p65 total levels, and reducing its nuclear translocation, upregulating Bcl2, and downregulating all measured apoptotic markers. Without any effect of apoptotic markers, α-LA also reduced the activities of NF-κB and levels of TNF-α in the livers of control rats, too, indicating potent independent anti-inflammatory and anti-apoptotic effects. Supporting this, α-LA inhibited TNF-α-induced adhesion molecules expression and activation of NF-κB in human aortic cells [67]. In the same line, α-LA prevented aflatoxin B1 and carbon tetrachloride (CCL_4_)-induced liver injury and angiotensin-II (ANG II)-mediated renal damage by suppressing NF-κB and the generation of TNF-α and IL-6 [68,69]. However, some authors have shown that the inhibitory effect of α-LA on NF-κB is an independent antioxidant mechanism that involves decreasing O-GlcNAcylation of NF-κB and suppressing the inhibitor of the nuclear factor kappa B, IκB [70,71]. However, α-LA administration reduced markers of apoptosis and upregulated levels of Bcl2 in the livers of AuNPs treated rats only with no effect on their expression in the control-treated rats. These data indicate that the anti-apoptotic effect of LA is secondary to its antioxidant and anti-inflammatory effects.

Targeting the keap-1/Nrf2 signaling pathway could provide a potential therapy against NPs-induced liver damage. Nrf2 plays a significant role in stimulating antioxidant genes and suppressing inflammation by inhibiting NF-κB [17,18,19,20]. On the other hand, studies have also shown a negative cross-talk between Nrf2 and NF-κB, where the latter can also repress Nrf2 in various mechanisms. Indeed, NF-κB p65 can repress Nrf2 nuclear localization and antioxidant transcriptional activity by the competitive interaction with the transcriptional coactivator CREB binding protein (CBP), which is required for the transcriptional activity of Nrf2 [19,72]. In addition, other studies have shown that the N-terminal area of the NF-κB p65 subunit is associated with an increased cytosolic keap-1 expression, leading to an increase in the degradation of Nrf2 in the cytoplasm and hence reduced nuclear levels [19,73]. Furthermore, p65 can induce nuclear translocation of keap-1, which also reduces the transcriptional activity of Nrf2 and subsequently reduces the expression of antioxidant genes [19,73].

In this study, we have targeted the keap-1/Nrf2/NF-κB axis in a trial to further explain the potential mechanism by which AuNPs and α-LA affect markers of oxidative stress and inflammation in the livers of rats. In addition, we aimed to understand the regulation between Nrf2 and NF-κB. In general, ROS are known to induce Nrf2 nuclear translocation and antioxidant gene expression by increasing the transcription of Nrf2, as well as by stimulating the phosphorylation of keap-1, which leads to a weak association with Nrf2 and hence its nuclear translocation [74]. Following and associated with the significant increase in the levels of ROS, TNF-α, and cytoplasmic/nuclear levels of NF-κB p65, the results of this study also revealed that treatment with AuNPs resulted in a significant reduction in the hepatic total cytoplasmic and nuclear protein levels of Nrf2. These data may explain the significant reduction in the levels of HO-1 and other antioxidants in the livers of AuNPs-treated rats, which is attributed to its inhibitory effect on Nrf2 translocation. To further explain the nuclear reduction in the levels of Nrf2, we have measured mRNA levels of both Nrf2 and its inhibitor, keap-1. Interestingly, no significant change in the levels of mRNA levels was seen in the livers of AuNPs-treated animals. Therefore, it sees very reasonable that AuNPs does not affect the transcription of keap-1 and Nrf2. Based on these data, we assumed that AuNPs reduce the total cytoplasmic and hence, the nuclear levels of Nrf2 by other indirect pathways such as strengthening the association between keap-1 and Nrf2 and activating NF-Κb P65, which, as discussed above, normally suppresses Nrf2 translocation. However, these mechanisms are still hypothetical and need further examination. Nonetheless, our data contradict those reported by Lai et al. [26], who have shown that AuNPs (3–5 nm) can induce Nrf2/HO-1 in cultured endothelial cells. This disparity can be explained by several factors, including the variation in the tissue targeted, the model used (animal modes vs. cultured cells), dose and size of AuNPs, and treatment period.

At this stage, the mechanism by which AuNPs repress Nrf2 remains unknown. However, several reports have shown that multiple factors may contribute to Nrf2 repression in tissue-specific manners [75]. In addition, Nrf2 signaling is controlled at the levels of translational levels without negligible change in mRNA levels of keap-1 and Nrf2 [76]. Interestingly, and independent of modulating keap-1 levels, Nrf2 activators can enhance the cytoplasmic and nuclear levels of Nrf2 by disabling the ubiquitination activity of keap-1 through interacting with its reactive cysteine residues [77]. In addition, dephosphorylating the serine 40 residue of Nrf2 increased the association between keap-1 and Nrf2, leading to degradation of Nrf2 and reducing its activation [78]. On the contrary, phosphorylation of keap1 at tyrosine 141 residue strengthens the association with Nrf2, leading to rapid degradation and reduced nuclear translocation of Nrf2 [79]. Additionally, GSK3β can stimulate the nuclear export and the cytoplasmic degradation of Nrf2 by direct phosphorylation of Nrf2 or Fyn, independent of keap-1 [80]. Therefore, it could be possible that AuNPs stimulate the degradation and repress the nuclear localization of Nrf2 by altering the activities of certain phosphatases and kinases, including GSK3β. This cannot be concluded from our data but could be further characterized by more deep studies. Despite this, our data still contradict some previous findings. In this context, AgNPs but not AuNPs significantly stimulated the nuclear activation of Nrf2 and the expression of HO-1 in the Caco-2 cell line (HTB-37) [81]. On the other hand, AuNPs activated Nrf2 signaling in the macrophages and keratinocytes by direct mechanism (binding to keap-1) or indirectly through increasing ROS and depleting GSH levels [27]. Additionally, AuNPs increased total and nuclear protein levels of Nrf2 and stimulated HO-I expression in human endothelial cells by either modification of keap-1 or Nrf2, in a ROS-dependent manner [26]. These were associated with normal mRNA levels of Nrf2 and keap-1. A contradiction between our results and those reported previously could be explained by the in vivo part of our study, the dose of AuNPs, and treatment periods.

Nevertheless, our data indicate that the protective effect of α-LA is mediated by stimulating Nrf2 signaling. Herein, the treatment with α-LA did not affect the levels of keap-1 nor mRNA levels of Nrf2 but significantly increased the total and nuclear levels of Nrf2 in the livers of control and AuNPs-treated rats. These data might indicate that the α-LA is acting by weakening the interaction between keap-1 and Nrf2 rather than affecting their transcription. These findings also support that typical Nrf2 activators stimulate Nrf2 without modulating keap-1 expression but by disabling the ubiquitination activity of keap1 (through interacting with its cysteine residues) or through stimulating some growth factors that inactivate GSK3β [77]. These data could explain why the livers of control and AuNPs-treated rats showed higher levels of GSH, SOD, and CAT and higher mRNA levels of HO-1. Supporting our data, the treatment with α-LA prevented HFD-induced hepatic steatosis by stimulating the nuclear localization of the Nrf2 [48,73]. It also prevented age-related loss of liver GSH by increasing the binding of Nrf2 to ARE [76]. In the same manner, α-LA attenuated methotrexate-induced hepatic cell activation by activating Nrf2 and subsequent upregulation of HO-1 [38].

In conclusion, the data of this study is the first to show that hepatic toxicity induced by AuNPs is associated with suppression of the Nrf2/antioxidant axis and increased levels and nuclear activation of NF-κB p65. In addition, it demonstrates the ability of α-LA to attenuate this hepatic toxicity of these NPs, at least by reversing these pathways, which subsequently ameliorated AuNPs-mediated liver oxidative stress, inflammation, and apoptosis. These data widen our knowledge about the toxic pathways underlying the toxic effect of AuNPs and provide effective alternative therapy.

## 5. Study Limitations

Some limitations still exist in this study. Importantly, this study examined the effect of AuNPs on the liver activity of NF-κB and Nrf2 using smaller size particles and after 7 days of treatment. Studying these effects at different time intervals (i.e., hours to days) will enrich the findings and reflect a complete comprehensive picture of the hepatotoxic effect of these particles. In addition, our data is still observational. Further studies using Nrf2 knock-down animals or hepatocytes are required to confirm these effects.

## Data Availability

The datasets used and analyzed during the current study are available from the corresponding author upon reasonable request.
